# Langerhans Cell Histiocytosis and Other Histiocytic Lesions

**DOI:** 10.1007/s12105-025-01766-2

**Published:** 2025-02-25

**Authors:** Reed A. McKinney, Guanghua Wang

**Affiliations:** 1 Head and Neck Pathology, The Joint Pathology Center, Silver Spring, MD USA; 2 Molecular Diagnostics Laboratories, The Joint Pathology Center, Bethesda, MD USA

**Keywords:** Langerhans Cell Histiocytosis, Erdheim-Chester Disease, Rosai-Dorfman-Destombes Disease, Cutaneous and Mucocutaneous Non-Langerhans Cell Histiocytosis, Hemophagocytic Lymphohistiocytosis, Malignant Histiocytoses, ALK-positive Histiocytosis, Touton Giant Cells, Emperipolesis, Mitogen-activated Protein Kinase (MAPK), *BRAF*, *MAP2K1*, *KRAS*, *NRAS*, *TP53*, *CDKN2A*

## Abstract

**Background:**

Histiocytoses, including Langerhans cell histiocytosis (LCH), comprise a diverse group of histiocytic disorders characterized by the abnormal accumulation and proliferation of histiocytes in various tissues or organs throughout the body, ranging from benign, self-limited conditions to aggressive malignancies and systemic inflammatory syndromes. These lesions present unique diagnostic challenges due to their broad spectrum of clinical presentations, overlapping histopathological and immunophenotypical features, and genetic complexity.

**Methods:**

This review analyzes major histiocytic lesions, focusing on their epidemiology, clinical presentations, histologic and immunophenotypic features, and genetic characteristics to facilitate accurate diagnosis and differentiation among these histiocytoses.

**Results:**

LCH, a well-recognized lesion, can affect various organ systems and necessitates differentiation from other types of histiocytoses such as Erdheim-Chester disease (ECD), Rosai-Dorfman-Destombes disease (RDD), and cutaneous and mucocutaneous non-Langerhans cell histiocytoses. Some histiocytic lesions, such as histiocytic sarcoma, are inherently malignant, while others, like hemophagocytic lymphohistiocytosis (HLH), manifest as severe, potentially life-threatening systemic inflammatory syndromes. Recent molecular genetic studies revealed recurrent genetic alterations in the MAPK pathway, such as *BRAF* V600E and *MAP2K1* in LCH and ECD, and *KRAS*, *NRAS*, and *MAP2K1* mutations in a subset of RDD. Malignant histiocytoses frequently show alterations in tumor suppressor genes like *TP53* and *CDKN2A*.

**Conclusion:**

Precise classification of histiocytic lesions relies on a comprehensive diagnostic approach that integrates clinical, histologic, immunophenotypic, and genetic data. Recent genetic advances shed light on these conditions’ unique but occasionally overlapping pathogenic mechanisms. Molecular genetics advancements continue to refine diagnostic accuracy and present new therapeutic targets, especially for aggressive or treatment-resistant cases.

## Introduction

Langerhans cell histiocytosis (LCH) and other histiocytic lesions encompass a diverse set of lesions characterized by the accumulation of macrophage, dendritic cell, or monocyte-derived cells in various tissues and organs across all age groups. These lesions are frequently accompanied by an inflammatory infiltrate rich in eosinophils. In line with evolving new findings regarding the cellular origins, molecular pathology, and clinical features of histiocytic disorders, based on histology, phenotype, molecular alterations, and clinical and imaging characteristics, the Histiocyte Society in 2016 classified histiocytoses into five main disease groups: (1) Langerhans cell related, (2) cutaneous and mucocutaneous, (3) malignant histiocytoses, (4) Rosai-Dorfman- Destombes disease (RDD), and (5) hemophagocytic lymphohistiocytosis and macrophage activation syndrome. This classification reflects the clinical behavior of these diseases, ranging from benign, self-limited conditions to aggressive malignancies and systemic inflammatory syndromes [1].

LCH, the most well-known of these disorders, is a clonal proliferation of Langerhans cells that can affect virtually any organ, most commonly the bones, skin, and lungs. The histopathologic hallmark of LCH is the presence of Langerhans cells with nuclear grooves and abundant eosinophilic cytoplasm, often accompanied by eosinophils, lymphocytes, and multinucleated giant cells. Immunohistochemically, LCH is defined by the expression of CD1a, S100, and langerin. Recent advances in genetic studies have shown that approximately 50–60% of LCH cases harbor the *BRAF* V600E mutation, while about 25% of cases demonstrate somatic *MAP2K1* mutations [2, 3]. Other histiocytic lesions, including Erdheim-Chester disease (ECD), Rosai-Dorfman-Destombes disease (RDD), cutaneous and mucocutaneous non-Langerhans cell histiocytoses, malignant histiocytoses (MH), hemophagocytic lymphohistiocytosis (HLH), and ALK-positive histiocytosis can show overlapping histological and clinical features, making differential diagnosis a significant challenge for pathologists. While these disorders share certain histopathological features, they exhibit distinct biological behaviors, ranging from benign self-limiting lesions to aggressive systemic diseases. The management and prognosis can vary widely depending on the underlying condition.

This article aims to provide a comprehensive overview of LCH and other histiocytic lesions, focusing on their epidemiology, clinical presentations, histopathological, immunohistochemical, and molecular features, as well as the critical elements of differential diagnosis. The goal is to offer practical insights for pathologists, facilitating accurate classification for optimal clinical management of these often complex and challenging cases.

## Langerhans Cell Histiocytosis

LCH is a clonal neoplastic proliferation of Langerhans cells expressing CD1a, langerin, and S100 protein and recurrent genetic alterations involving RAS/RAF/MEK/ERK pathway.

### Epidemiology

LCH remains a rare condition, with an estimated incidence of 2 to 10 cases per million children aged 15 years or younger. There is an approximal equal distribution in male and female patients [4, 5]. Based on data from the Surveillance, Epidemiology, and End Results program for 2010 to 2016, the incidence of LCH was highest in the first year of life and then declined steadily with age. Beyond the age of 21, the rate of cases remains relatively stable across the adult age range [6].

### Clinical Presentation

**Fig. 1:**
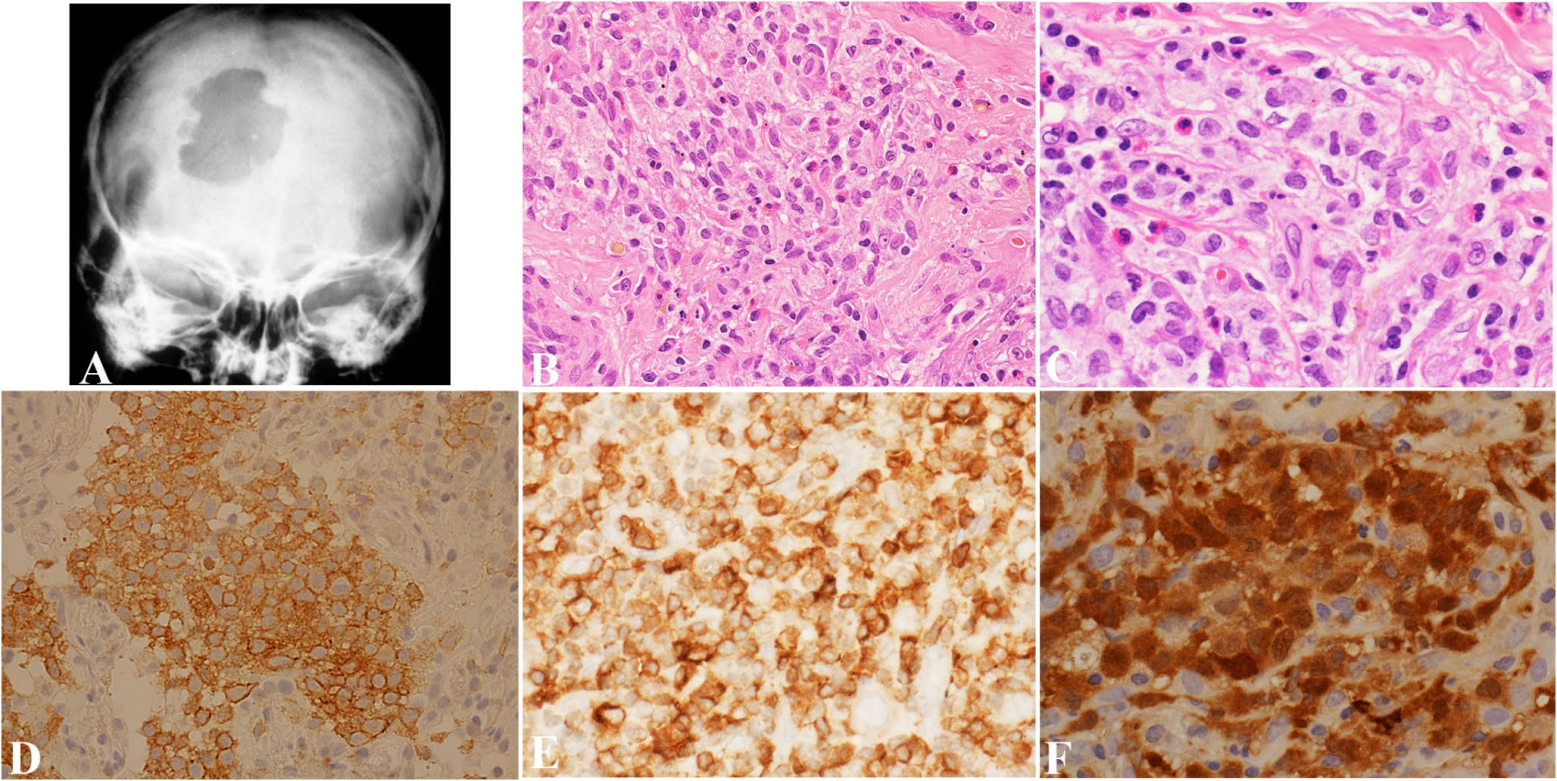
Langerhans cell histiocytosis. (**A**) A skull radiograph shows the typical appearance of lytic lesions of Langerhans Cell Histiocytosis in bone. The lesion exhibits scalloped edges and sharp borders with a radiodense focus that is commonly seen in skull lesions. Image from Wang G. Langerhans Cell Histiocytosis, in DP: Molecular Oncology, 3e, by Vasef M, et al. Copyright Elsevier 2024. Reused with permission. (**B**) The H&E section shows a sheet of oval-shaped Langerhans cells with eosinophilic cytoplasm, linear grooves, and inconspicuous nucleoli. Abundant eosinophils are present. (**C**) High-power view highlights the typical nuclear features of Langerhans cells. The nuclei are folded, show linear grooves, and often resemble coffee beans. (**D**) Immunostain CD1a diffusely marks Langerhans cells with positive membranous and cytoplasmic immunoreactivity. (**E**) Langerhans cells show a cytoplasmic granular staining pattern with langerin. (**F**) Langerhans cells show positive cytoplasmic and nuclear staining with S100

Childhood LCH varies clinically, from mild, single-organ involvement to severe, multisystem disease with symptoms ranging from asymptomatic bone lesions to life-threatening systemic illness [7, 8]. The skeleton is most affected, with up to 80% of cases presenting with bone lesions, which can appear radiographically as lytic (Fig. 1A) or widespread [9–11].

In adult LCH, most of the clinical and radiographic findings are like those of childhood LCH; however, isolated skin lesions are uncommon in adults. Radiographic bone lesions are present in 30–50% of cases, frequently involving the skull and dental sites, and less frequently pelvis, vertebrae, ribs, and extremities with the characteristic “punched-out” bony lesions [12]. Lung involvement in LCH can be part of multisystem disease or, more commonly in adults, can present as a primary or predominant form known as pulmonary LCH (PLCH). PLCH is a rare diffuse cystic lung disease, typically affecting young to middle-aged adults of both sexes and is seen almost exclusively in smokers or former smokers [13].

### Pathogenesis

The etiology of LCH has been a subject of significant research, evolving from theories of reactive processes to its current classification as a clonal neoplastic disorder. Langerhans cells are specialized, bone marrow-derived dendritic cells that act as antigen-presenting cells in the epidermis and mucosal tissues. They are uniquely identified by the markers CD1a, S100, and langerin (CD207) and contain distinctive Birbeck granules (“tennis racket” structures). Research suggests that circulating CD1c⁺ myeloid dendritic cells (mDCs) may serve as precursors to the pathogenic CD1a⁺CD207⁺ cells in LCH and, once activated via the ERK pathway, migrate from the bloodstream to lesion sites [14].

The neoplastic nature of LCH was first suggested by evidence of clonality in Langerhans cells through X-chromosome inactivation analysis. The presence of clonal histiocytes in all forms of LCH supports that it is a clonal neoplastic disorder rather than a reactive process with highly variable biological behavior [15].

The discovery of somatic mutations in the mitogen-activated protein kinase (MAPK) signaling pathway, especially *BRAF* V600E, has been key to understanding LCH pathogenesis. *BRAF* V600E, the most common mutation in LCH, occurs in 50–60% of cases and leads to continuous MAPK pathway activation, driving uncontrolled Langerhans cell proliferation [2]. In addition to *BRAF*, *MAP2K1* mutations are found in 20–30% of LCH cases lacking *BRAF* mutations. Mutations in *MAP2K1*, which encodes the protein MEK1, lead to the constitutive activation of the pathway, promoting the uncontrolled proliferation of Langerhans cells—a hallmark of LCH [3, 16, 17]. *MAP2K1* and *BRAF* mutations are mutually exclusive. Mutually exclusive somatic activating mutations in MAPK pathway genes have now been identified in approximately 85% of LCH lesions [18]. These mutations, more prevalent in aggressive forms of LCH, underscore the disease’s neoplastic nature and serve as targets for therapy [1].

While genetics is central to LCH pathogenesis, environmental and immune factors, such as viral infections (e.g., EBV) and exposures like tobacco smoke, are proposed contributors, though their precise roles remain unclear.

Immunological dysregulation significantly impacts LCH pathogenesis, with abnormal Langerhans cell proliferation accompanied by an inflammatory milieu of T cells, B cells, macrophages, and eosinophils, which suggests an immune response to the neoplastic cells. These immune cells, particularly T cells, secrete cytokines (e.g., IL-17, IL-6, and TNF-α) that contribute to the persistence of the lesions. The release of pro-inflammatory cytokines further promotes the abnormal proliferation of LCH cells and drives tissue destruction. Increased regulatory T cells (Tregs) and M2 macrophages in lesions further suppress the immune response, supporting LCH cell survival and lesion chronicity [19].

Eosinophils are a significant inflammatory component in LCH lesions, but their recruitment mechanisms and functional role in the disease remain poorly understood. An early study suggests that eosinophil recruitment may be mediated by CCL5 produced by Langerhans cells, but this finding has yet to be widely validated [20]. The precise contribution of eosinophils to LCH pathogenesis is not yet fully defined, underscoring the need for additional research to clarify their role in LCH.

Immune evasion is observed in LCH. Neoplastic LCH cells often express PD-L1, an immune checkpoint protein that interacts with PD-1 on T cells, leading to an inhibited immune response and allowing LCH cells to evade destruction [21]. This immune-tolerant environment supports the persistence of LCH cells and contributes to the chronicity of the disease.

### Diagnosis

Diagnosing LCH involves clinical assessment, histopathology, immunohistochemistry, molecular testing, and imaging to accurately identify the disease and assess its extent. A comprehensive evaluation of LCH has been outlined by Haupt et al. [22] Definitive diagnosis is based on pathology, with diagnostic Langerhans cells showing ovoid shape, grooved nuclei, fine chromatin, and minimal nuclear atypia (Fig. 1B-C); in some cases, the Langerhans cells have an increased Ki-67 proliferation index and mitotic activity. Along with Langerhans cells, a characteristic mixed inflammatory infiltrate is commonly seen, consisting of eosinophils, histiocytes, neutrophils, plasma cells, and small lymphocytes. Eosinophil-rich inflammatory infiltrates may also be seen in various conditions, such as allergic reactions, infections, and some malignancies. These add another layer of complexity to the diagnostic process and often require careful correlation with clinical, radiologic, and immunohistochemical findings to prevent misdiagnosis.

Langerhans cells stain positively for CD1a and langerin (Fig. 1D-E) as well as S100, showing both cytoplasmic and nuclear staining patterns (Fig. 1F), while markers like CD1c, B-cell markers, T-cell markers, CD30, and CD23 are negative. The *BRAF* V600E mutation-specific antibody (VE1) is positive in many cases, along with vimentin, CD4, and CD68, while CD163 positivity is only observed in 5–10% of cases. Immunohistochemistry for phosphorylated ERK (pERK) can also indicate MAPK pathway activity but is less specific than genetic testing [21]. On electron microscopy, Birbeck granules may appear but are not essential for diagnosis.

Molecular testing has dramatically advanced diagnosing and treating LCH by identifying genetic mutations in the MAPK pathway. Approximately 50–60% of LCH cases have the *BRAF* V600E mutation, which drives abnormal cell growth, while *MAP2K1* mutations are found in an additional 20–30%, especially in *BRAF*-negative cases [21, 23]. Next-generation sequencing (NGS), Sanger sequencing, and pyrosequencing are standard methods for detecting these mutations. Use of these molecular tests significantly enhances the accurate diagnosis of this entity.

### Treatment

Recent international expert consensus recommendations outline treatment options for adult LCH that vary based on disease extent, organ involvement, and genetic profile [12]. For patients with single-system LCH, surgery is often curative, with radiation as an alternative for inoperable lesions. Vinblastine and prednisone are standard first-line treatments for multisystem LCH, with more intensive chemotherapy reserved for high-risk organ involvement. Targeted therapies, including BRAF inhibitors (e.g., vemurafenib) for *BRAF* V600E mutations and MEK inhibitors (e.g., trametinib) for *MAP2K1* mutations, show promise in refractory cases [21]. Supportive care, such as hormone replacement for pituitary involvement, is essential for quality of life.

### Prognosis

The prognosis of LCH depends on disease extent, organ involvement, genetic mutations, and the responses to treatment. The prognosis in childhood LCH is guarded, as it is determined by the response to the initial therapeutic interventions, age of diagnosis, and extent of organ involvement. No response to initial therapy indicates a poor prognosis [24]. Unifocal LCH has a 5-year overall survival rate of over 90%. Multisystem LCH involving high-risk organs like the liver, spleen, or bone marrow has a more guarded prognosis, requiring systemic therapies such as chemotherapy or targeted treatments. Outcomes vary based on treatment response and specific organ involvement, with adult prognosis often affected by treatment efficacy and relapse frequency. Prognosis can range from no sequelae to severe organ dysfunction (e.g., liver or lung) or neurodegeneration. The presence of *BRAF* V600E mutations, often seen in aggressive LCH, correlates with higher relapse rates and multisystem disease. Targeted therapies, including BRAF and MEK inhibitors, have improved outcomes in high-risk patients and those unresponsive to standard treatments [25].

## Erdheim-Chester Disease

ECD is a clonal proliferation of histiocytes characterized by the infiltration of tissues by foamy histiocytes and Touton giant cells.

### Epidemiology

ECD is a rare, likely underdiagnosed condition, with an estimated 1,500 cases reported globally so far. Approximately 70% of cases occur in men, commonly diagnosed in adults between the ages of 40 and 60 years, with a mean age of 50 years. Cases in pediatric patients are rare, and they are frequently associated with LCH. Approximately 20% of ECD patients have an LCH lesion; it may also be seen in the same biopsy [26].

### Clinical Presentation

**Fig. 2:**
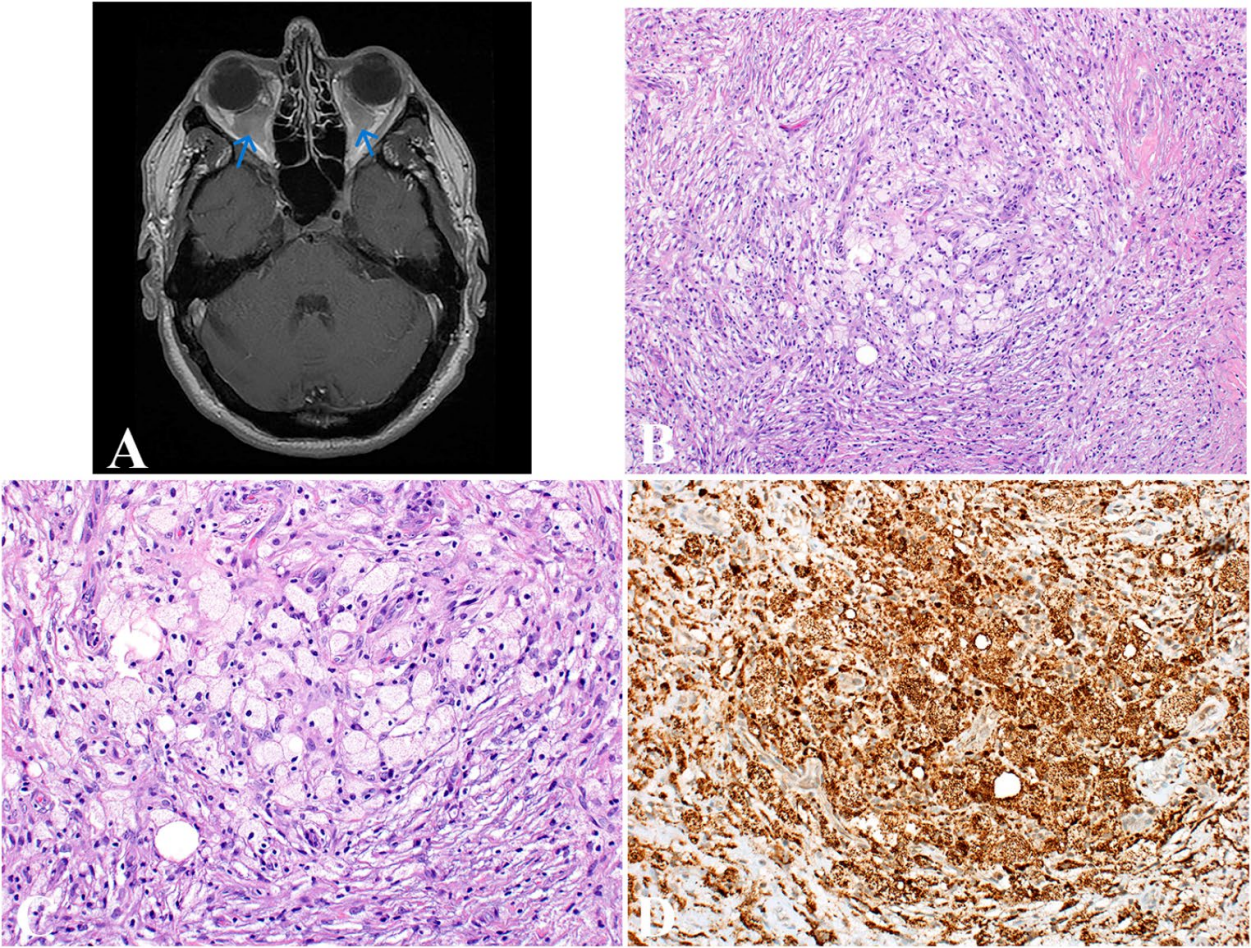
Erdheim-Chester disease. (**A**) T1-weighted axial magnetic resonance image shows bilateral proptosis secondary to diffuse replacement of the intraconal fat by lesional tissue that encases the optic nerves marked with blue arrows. (**B**) Low-power view shows collections of foamy histiocytes with fibrosis. (**C**) High-power view highlights foamy histiocytes with abundant, lipid-rich cytoplasm and small nuclei. (**D**) Immunostain CD68 diffusely marks foamy histiocytes

ECD is a systemic histiocytic neoplasm characterized by multiorgan proliferation of histiocytes in a fibrotic background. Bilateral symmetrical osteosclerosis of the long bones of the lower extremities is characteristic, occurring in up to 95% of cases [27]. CD1a-negative histiocytes deposit in the bone, kidneys, retroperitoneum, heart, lungs, skin, and brain; however, the lymph nodes, spleen, and liver are generally spared [28]. Lesional histiocytes can infiltrate retrobulbar soft tissue in the orbits, leading to exophthalmos and visual disturbances (Fig. 2A). Endocrine complications, such as diabetes insipidus, are common, alongside neurological symptoms like ataxia or cognitive changes, which occur in about 30–50% of cases. Cardiovascular complications (e.g., pericardial infiltration and “coated aorta”) and retroperitoneal fibrosis (e.g., “hairy kidney” appearance) are also significant markers of ECD. Patients with ECD present with an autoimmune condition 41% of the time, commonly in the form of thyroiditis, Sjögren syndrome, systemic lupus erythematous, pernicious anemia, or polymyalgia rheumatica [27].

### Pathogenesis

The etiology is primarily linked to somatic mutations in genes in the MAPK pathway. The *BRAF* V600E mutation is the most common genetic alteration, occurring in approximately 50–60% of patients with ECD. Other mutations affecting the MAPK pathway such as *MAP2K1* and *ARAF* have also been identified in ECD patients. These mutations lead to constitutive activation of the MAPK/ERK signaling pathway, resulting in uncontrolled cell proliferation and survival [29]. In another study, 10.9% of patients presented with a *PIK3CA* mutation and 3.7% with an *NRAS* mutation [30].

### Diagnosis

The diagnosis of ECD involves clinical, radiological, histopathological, and genetic evaluations. Imaging, particularly PET-CT scans, plays a key role in diagnosis, showing symmetric long bone sclerosis, cardiovascular infiltration, or retroperitoneal fibrosis, which are characteristic findings in ECD [31]. A biopsy is required for the definitive diagnosis of ECD. The hallmark of ECD is the presence of foamy histiocytes with abundant, lipid-rich cytoplasm and small nuclei. These histiocytic infiltrates are often surrounded by fibrosis and inflammatory infiltrates, including lymphocytes, plasma cells, and neutrophils, but eosinophils are rare to absent (Fig. 2B-C). Multinucleated histiocytes or Touton giant cells are commonly encountered [29]. Although not common, emperipolesis may also be present in ECD. Immunohistochemical analysis reveals that ECD histiocytes are typically positive for CD68, CD163, and factor XIIIa (Fig. 2D). Some histiocytes may be positive for S100 protein but negative for CD1a and langerin, which differentiates ECD from LCH [32]. Testing for *BRAF* V600E and other MAPK pathway mutations (*MAP2K1* variants) is essential in ECD, as 50–60% of patients have the *BRAF* V600E mutation. Testing for *BRAF* V600E and *MAP2K1* mutations is especially valuable in challenging cases where ECD may be confused with extracutaneous or disseminated juvenile xanthogranuloma because characteristic genetic aberrations favor the diagnosis of ECD.

### Treatment

The treatment for ECD depends on the underlying genetic mutations and extent of disease, incorporating targeted therapies and supportive care. For patients with the *BRAF* V600E mutation, BRAF inhibitors (vemurafenib and dabrafenib) are effective with high response rates. In cases without *BRAF* mutations but with other MAPK pathway mutations (e.g., *MAP2K1*), MEK inhibitors (cobimetinib and trametinib) are alternatives. These inhibitors effectively target downstream MAPK elements, aiding in control, particularly for central nervous system involvement. In patients lacking targetable mutations or who cannot tolerate targeted therapies, interferon-alpha or cladribine can be used, though they are generally second-line options [31].

### Prognosis

The prognosis of ECD largely depends on the sites of involvement, the extent of organ involvement, the presence of genetic alterations in the MAPK pathway, and the patient’s response to treatment. ECD is a chronic, progressive disease; while many cases exhibit a slow progression, severe multisystem involvement—particularly of the cardiovascular, central nervous, and renal systems—can be life-threatening [31]. The introduction of targeted therapies, especially BRAF and MEK inhibitors, has significantly improved survival outcomes, with high response rates and better disease control [31]. For patients without actionable mutations, conventional therapies and supportive care remain essential, though the prognosis may be less favorable.

## Cutaneous and Mucocutaneous Non-Langerhans Cell Histiocytoses

Cutaneous and mucocutaneous histiocytoses refer to a diverse group of non-Langerhans cell (non-LCH) histiocytoses primarily affecting the skin and mucous membranes. These conditions range from benign, self-limited forms to more aggressive variants, with presentations including papules, nodules, or plaques. Common types include juvenile xanthogranuloma (JXG) and adult xanthogranuloma (AXG).

### Epidemiology

Cutaneous and mucocutaneous non-LCH histiocytoses include a wide variety of conditions affecting both children and adults. Precise incidence rates are unavailable for these disorders. Some subtypes, such as JXG, predominantly occur in children, whereas AXG and Necrotic Xanthogranuloma (NXG) are more commonly seen in adults [1]. Overall, there is no significant sex predominance across these types.

### Clinical Presentation

Cutaneous and mucocutaneous histiocytoses primarily affect the skin and mucous membranes, ranging from isolated benign lesions to more widespread forms, and are classified by site, age, and disease extent (solitary, localized, or disseminated). They fall under the “C” group of histiocytoses, including common lesions like juvenile and adult xanthogranulomas, and rarer forms such as solitary reticulohistiocytoma; benign cephalic histiocytosis; generalized eruptive histiocytosis; progressive nodular histiocytosis; cutaneous RDD and necrobiotic xanthogranuloma, two non-LCH histiocytoses with a major systemic involvement; xanthoma disseminatum; and multicentric reticulohistiocytosis [1].

### Pathogenesis

The etiology of cutaneous and mucocutaneous histiocytoses is unclear. While some histiocytoses, such as JXG, are thought to result from reactive immune processes rather than genetic causes, the exact triggers are not well understood. Limited evidence points to MAPK pathway mutations (e.g., *BRAF* and *MAP2K1*) in specific histiocytic disorders, though these are more commonly implicated in systemic rather than isolated cutaneous forms. The diversity within these conditions suggests a complex interplay of immune, environmental, and potentially genetic factors, and a definitive cause has not yet been established [1, 33].

### Diagnosis

**Fig. 3:**
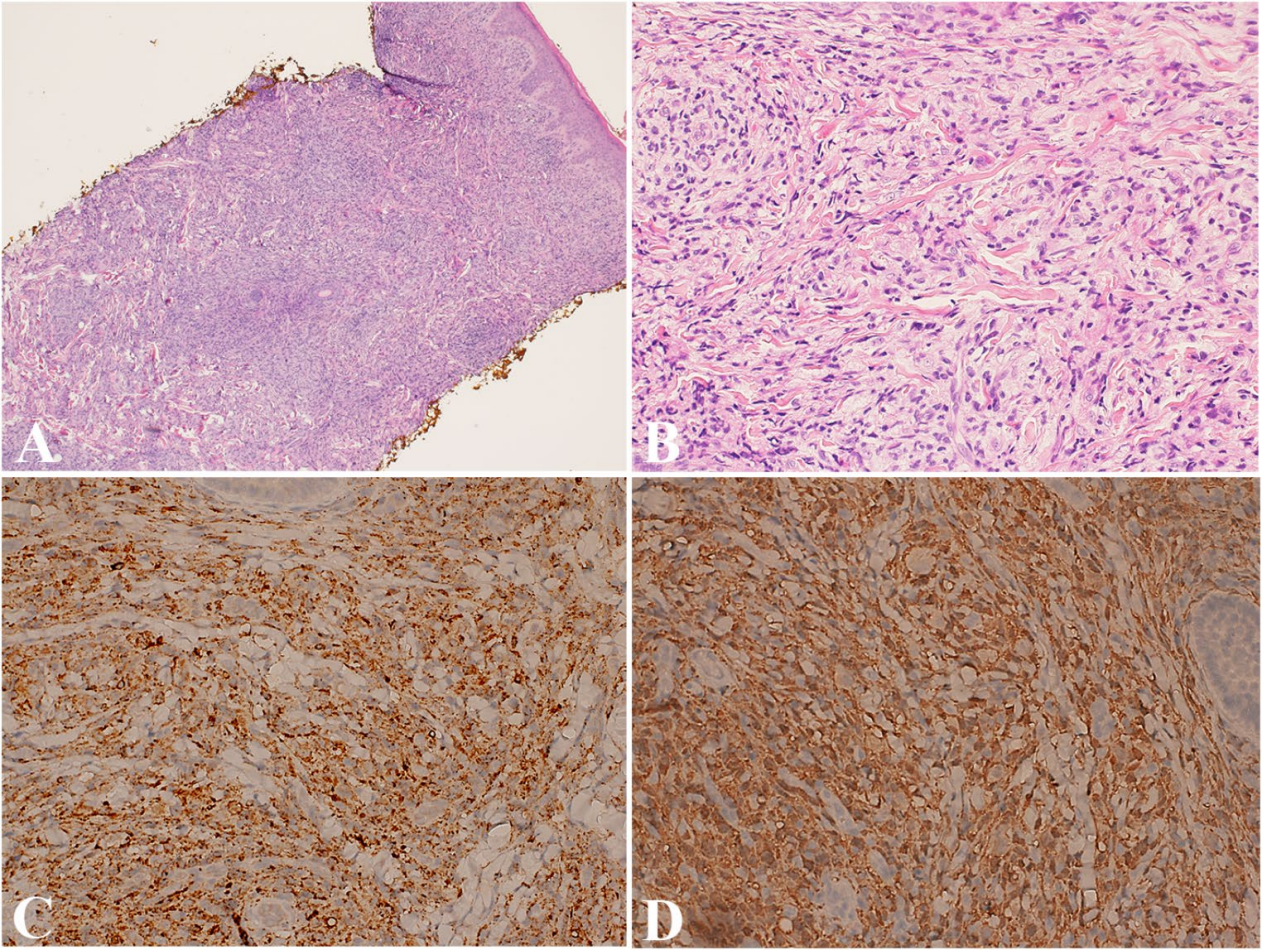
Juvenile xanthogranuloma. (**A**) Low-power view shows a proliferation of spindle cells in the dermis, sparing epidermis. (B) High-power shows more details of the proliferation of histiocytes arranged in short, interweaving fascicles that weave between collagen bundles. (**C**) Immunostain CD68 diffusely marks these histiocytes. (**D**) Immunostain factor XIIIa diffusely marks these histiocytes

Diagnosing cutaneous and mucocutaneous histiocytoses involves clinical evaluation, histopathology, and immunohistochemistry to differentiate them from other histiocytic or dermatologic conditions. Histologically, JXG and AXG present as well-circumscribed nodules in the dermis, sparing the epidermis, and feature foamy cells, giant cells, and Touton giant cells, often accompanied by eosinophils, macrophages, and lymphocytes (Fig. 3A-B). Over time, lesions typically undergo fibrosis. Solitary reticulohistiocytoma (SRH) also presents as a dermal nodule, resembling a xanthogranuloma composed of large histiocytes and multinucleated giant cells with abundant eosinophilic or ground-glass cytoplasm [1]. Immunohistochemical staining confirms their histiocytic nature, with positive CD68, CD163, and factor XIIIa (Fig. 3C-D) and negative CD1a, distinguishing JXG, AXG, and SRH from LCH. Other rarer cutaneous and mucocutaneous histiocytoses, such as benign cephalic histiocytosis, generalized eruptive histiocytosis, and xanthoma disseminatum, may show unique clinical and histological features, often with overlapping immunophenotypes including positive CD68, CD163, and factor XIIIa [34].

In addition, given the appropriate clinical scenario, special stains (e.g., PAS-D and AFB) for microorganisms to exclude a dermal fungal or mycobacterial infection may be indicated.

### Treatment

Treatment varies based on disease extent and severity. For patients with limited or isolated skin involvement, observation is often adequate, as many cases (e.g., JXG) resolve spontaneously. In symptomatic or cosmetically concerning cases, topical corticosteroids or surgical excision may be used [34]. For extensive skin involvement or cases with systemic symptoms, systemic treatments are often necessary. Corticosteroids are the mainstay, particularly for reducing inflammation. Immunosuppressive agents like methotrexate or vinblastine are used in cases with significant spread or resistance to initial treatment [33].

### Prognosis

The prognosis of cutaneous and mucocutaneous histiocytoses varies by subtype and extent of disease involvement. Overall, prognosis is highly favorable for localized, self-limited forms but varies for more widespread or recurrent disease, depending on systemic involvement and treatment response [33].

## Rosai-Dorfman-Destombes Disease

RDD is a histiocytic disorder characterized by the proliferation of large S100-positive histiocytes with conspicuous emperipolesis (lymphocytophagocytosis) of lymphocytes, plasma cells, or neutrophils.

### Epidemiology

RDD usually involves lymph nodes but also frequently involves extranodal sites. The cervical lymph nodes are the most commonly affected nodal site in RDD, typically presenting as bilateral, painless, massive cervical lymphadenopathy. Other nodal regions frequently involved include the mediastinal, inguinal, and retroperitoneal lymph nodes. Extranodal involvement is also a prominent feature of RDD, with commonly affected sites including the skin, nasal cavity, bones, soft tissues, retro-orbital tissues, gastrointestinal tract, kidneys and genitourinary tract, and respiratory system. It often affects young people (mean age, 30 years), and occurs more commonly in people of African descent, with a slight male predominance in this subset of affected individuals [35].

### Clinical Presentation

RDD is categorized primarily into three forms based on clinical presentation and associations [35–37].

Sporadic RDD (non-cutaneous) is the most common form of all RDDs. It encompasses several subtypes, classified based on the site of involvement and their association with other conditions, including classic nodal RDD, extranodal RDD, neoplasia-associated RDD, and autoimmune disease-associated RDD. Additional details are provided in Table 1.

**Table 1:** Classification of sporadic RDD

Subtype of Sporadic RDD	Description	Associated Conditions
Classic Nodal RDD	Involves lymph nodes, primarily presenting as massive, painless cervical lymphadenopathy	Lymphadenopathy
Extranodal RDD	Affects extranodal sites, including skin, bones, soft tissue, and retro-orbital tissues	Skin lesions, bone involvement, soft tissue masses, retro-orbital abnormalities
Neoplasia Associated RDD	Occurs in conjunction with malignancies	Lymphomas, leukemias, malignant histiocytosis
Immune-Disease Associated RDD	Linked to autoimmune conditions	Lupus erythematous, idiopathic juvenile arthritis, autoimmune hemolytic anemia

Familial RDD, linked to genetic syndromes, is rare and includes conditions such as H syndrome (Faisalabad Syndrome) and FAS deficiency-related RDD, also referred to as autoimmune lymphoproliferative syndrome-related RDD (ALPS-related RDD). H syndrome is caused by mutations in the *SLC29A3* gene and is characterized by nodal and extranodal RDD, along with features such as hyperpigmentation, short stature, and hypertrichosis. FAS deficiency-related RDD is a rare form of RDD that is associated with mutations in the *TNFRSF6* gene, which plays a critical role in regulating apoptosis. Patients with ALPS often present with systemic lymphoproliferation, autoimmune manifestations, and increased susceptibility to infections, reflecting broader immune dysregulation. FAS deficiency-related RDD is typically observed in more severe cases of ALPS patients.

Cutaneous RDD, classified separately under the “C group” of histiocytoses, predominantly affects the skin, presenting as papules or nodules, and is more frequent in older adults [1]. Unlike nodal RDD, cutaneous RDD generally lacks systemic involvement and has a more favorable course​ [35].

### Pathogenesis

The etiology of RDD remains partially understood and appears to involve both immune dysregulation and potential neoplastic processes. While RDD was previously considered a reactive disorder due to immune abnormalities and associations with autoimmune conditions, recent discoveries have identified somatic mutations in the MAPK pathway (*KRAS*, *NRAS*, *MAP2K1*, and *ARAF*) and activating mutations in *CSF1R* in some cases, suggesting a neoplastic origin in subsets of RDD [18, 35]. These mutations indicate a clonal process in certain cases, while others may arise from an immune-driven reaction to infectious or inflammatory triggers.

### Diagnosis

The diagnosis of RDD requires a combination of clinical evaluation, histopathology, and immunohistochemistry. RDD often presents with massive, painless lymphadenopathy, particularly in the cervical lymph nodes, and may include extranodal manifestations in the skin, central nervous system, respiratory tract, and other sites. Radiologic imaging, particularly MRI and CT scans, helps assess the extent of extranodal disease, especially in cases with central nervous system or retroperitoneal involvement.

**Fig. 4:**
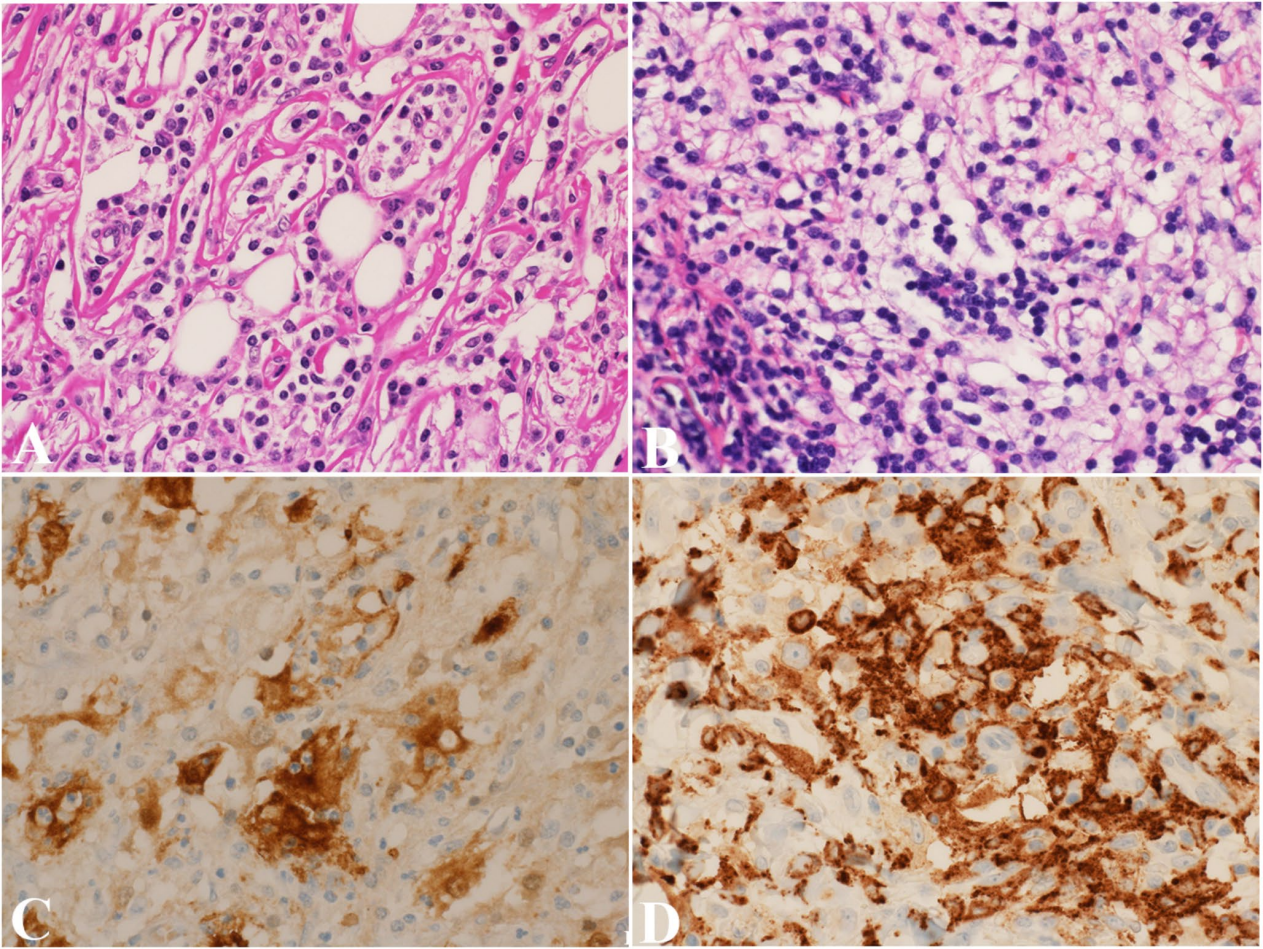
Rosai-Dorfman-Destombes disease. (**A**) Dense lymphohistiocytic infiltrate with plasma cells and areas of fibrosis. Scattered large histiocytic cells show significant emperipolesis. (**B**) Another case shows collections of histiocytes displaying emperipolesis. (**C**) Immunostain S100 is positive and outlines emperipolesis. (**D**) Immunostain CD163 is diffusely positive

A tissue biopsy is essential for diagnosing RDD. Microscopically, RDD lesions are characterized by collections of histiocytes with large histiocytes displaying emperipolesis—engulfment of intact lymphocytes, plasma cells, and neutrophils within the histiocyte cytoplasm (Fig. 4A-B). Though nonspecific, this feature is a hallmark of RDD. A chronic inflammatory infiltrate with lymphocytes and plasma cells with rare or no eosinophils is commonly seen between collections of histiocytes. On low magnification, a striated appearance of the light and dark zones is appreciated. Immunohistochemically, RDD histiocytes are typically positive for S100, CD68, and CD163, but CD1a and langerin are negative, helping distinguish RDD from LCH and other histiocytic disorders (Fig. 4C-D) [35]. BCL1 positivity may aid in the diagnosis, particularly in extranodal presentations.

Molecular testing for genetic alterations in the MAPK pathway is used to aid the diagnosis, and it is especially useful in refractory cases or when targeted therapy is considered. Approximately 50% of RDD lesions harbor mutations in genes in the MAPK/ERK pathway, including *KRAS*, *NRAS*, *MAP2K1*, *ARAF*, *CSF1R*, and rarely *BRAF* V600E [35, 37].

### Treatment

The RDD treatment is customized based on disease severity and extent. Mild, uncomplicated, or asymptomatic cases typically require observation and monitoring, while localized disease may benefit from surgical removal of affected lymph nodes or masses. Systemic therapies, including corticosteroids, sirolimus, chemotherapy, immunomodulatory therapy, targeted therapies, and radiotherapy, are considered for unresectable diseases [36]. In 2022, the U.S. Food and Drug Administration (FDA) approved Cotellic (cobimetinib) for the treatment of adults with histiocytic neoplasms, including RDD [38].

### Prognosis

The prognosis of RDD varies based on disease extent and organ involvement. Most cases of classic nodal RDD are self-limited and have a favorable prognosis, often requiring minimal intervention. Extranodal or systemic forms, especially with central nervous system or respiratory involvement, may follow a more chronic and relapsing course, impacting quality of life and leading to potential complications [36]. The development of targeted therapies for patients with RDD harboring MAPK pathway mutations has improved outcomes in refractory cases, offering more effective disease control.

## Primary and Secondary Malignant Histiocytoses

Malignant histiocytoses (MH) are a rare, aggressive group of malignant neoplasms originating from histiocytes or dendritic cells, characterized by the expression of histiocyte/dendritic cell markers such as CD68, CD163, CD4, and lysozyme [1]. The diagnosis requires ruling out other tumors, confirmed by the absence of immunohistochemistry markers like keratins, EMA, Melan-A, HMB45, B and T lymphocyte markers, and follicular dendritic cell markers. These neoplasms are classified into primary and secondary forms [1].

### Epidemiology

Both primary and secondary MH are extremely rare. Due to their rarity, epidemiological data are sparse, and diagnosis is usually confirmed in specialized medical centers with advanced diagnostics technologies.

### Clinical Presentation

Primary MH include histiocytic sarcoma, Langerhans cell sarcoma, interdigitating dendritic cell sarcoma, and indeterminate dendritic cell tumor. They often involve lymph nodes and extranodal sites like the liver, spleen, and gastrointestinal tract and often present with nonspecific symptoms, such as fever, weight loss, fatigue, lymphadenopathy, and hepatosplenomegaly, with possible gastrointestinal, skin, or bone involvement causing localized symptoms.

Secondary malignant histiocytosis generally transforms from conditions like chronic lymphocytic leukemia, diffuse large B-cell lymphoma, or follicular lymphoma and presents similarly, with systemic symptoms and possible extranodal spread [39].

### Pathogenesis

Primary MH are de novo malignancies arising from histiocytes or dendritic cells; there is no well-defined etiology, but MH are possibly linked to genetic mutations affecting cell differentiation and proliferation. Mutations in *CDKN2A*, *BRAF*, *KRAS*, *NRAS*, *TP53*, *MAP2K1*, *NF1*, *PTPN11*, and *CSF1R* have been identified in some primary MH lesions, contributing to unregulated cell growth [18, 40–42]. A study of genomic profiling of primary histiocytic sarcoma suggests the existence of a distinct subtype of primary histiocytic sarcoma characterized by *NF1/PTPN11* alterations with predilection for the gastrointestinal tract [42]. The identification of pathogenic genetic mutations suggests a role in pathogenesis. However, the exact mechanisms and risk factors contributing to the development of primary malignant histiocytosis are poorly defined, warranting further research [18].

Secondary MH results from anaplastic progression with aberrant expression of histiocyte/dendritic cell markers or transdifferentiation of an existing hematologic proliferation, particularly lymphomas and leukemias. These cases retain some molecular characteristics of the original malignancy, such as immunoglobulin gene rearrangements, the t(14;18) translocation, *BRAF* mutations, or other chromosomal alterations, distinguishing them from primary histiocytic neoplasms [1, 43].

### Diagnosis

**Fig. 5:**
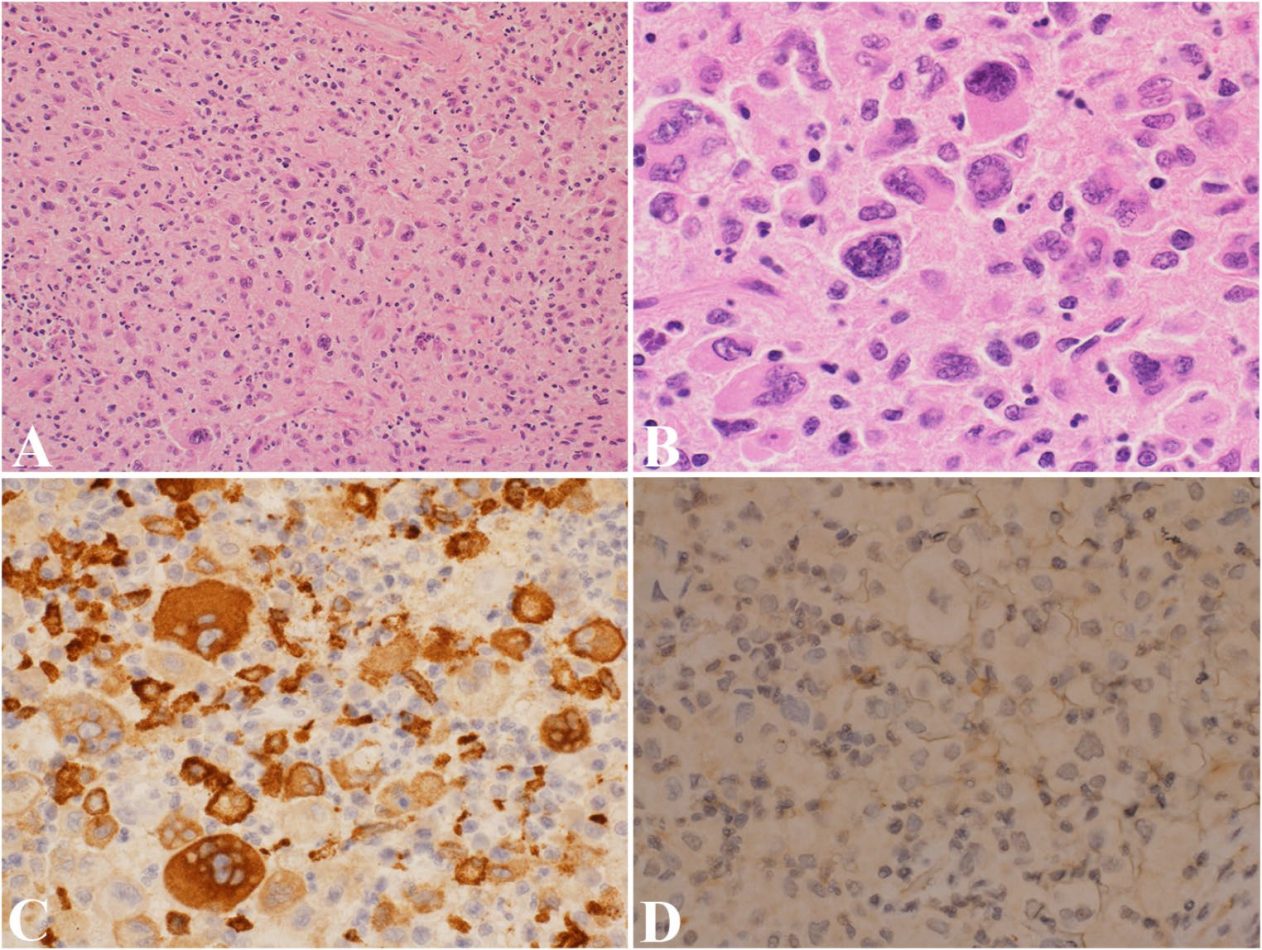
Malignant histiocytosis. (**A**) Sheets of polygonal cells with abundant eosinophilic cytoplasm and pleomorphic nuclei with prominent nucleoli. (**B**) High-power view reveals an anaplastic appearance of malignant histiocytes with marked cytologic atypia. (**C**) Immunostain CD163 highlights malignant tumor cells. (**D**) CD1a is negative

As with the other histiocytic diseases, a combination of clinical, radiographic, and histologic phenotypes contributes to the diagnosis of a malignant process. Microscopically, MH is characterized by anaplastic histology consisting of histiocytic cells with large pleomorphic nuclei containing vesicular chromatin and distinct nucleoli, and abundant eosinophilic cytoplasm with significant mitotic activity with atypical mitoses (Fig. 5A-B). These histopathological features are mandatory for diagnosis. Immunohistochemistry studies are needed to exclude other tumors in the differential diagnosis by showing negativity for keratins, EMA, Melan-A, HMB45, B and T lymphocyte markers, follicular dendritic cell (DC) markers, and expression of at least two of the following histiocyte/DC markers: CD68, CD163, CD4, and lysozyme (Fig. 5C) [1]. A recent study by Ravindran et al. shows that all malignant histiocytic lesions express CD68 and BCL1 [44]. MH can be categorized into four types of lineage differentiation: macrophages, monocytes, dendritic cells, and Langerhans cells [44].

**Fig. 6:**
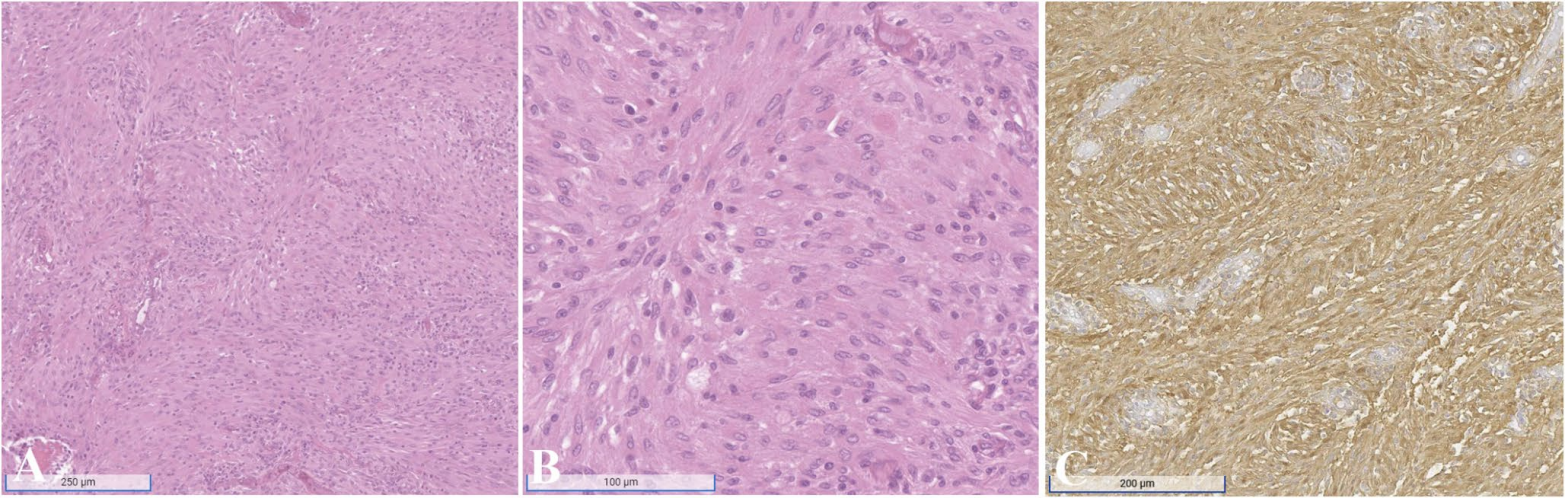
Interdigitating dendritic cell sarcoma. (**A**) Low-power view reveals sheets of spindle cells arranged in fascicles with a whorled pattern. (**B**) High-power view reveals tumor cells with abundant, slightly eosinophilic cytoplasm lacking distinct cell borders. (**C**) S100 protein is diffusely positive

Given it overlapping features with MH, melanoma should be excluded using melanocytic markers such as SOX10 and HMB45, which are typically negative in MH. Histiocytic sarcoma presents with large, pleomorphic histiocytic cells, which are positive for CD68, CD163, lysozyme, and CD4 and negative for CD1a (Fig. 5D), langerin, MPO, and SOX10. Histiocytic sarcoma should be differentiated from diffuse large B-cell lymphoma or T-cell lymphoma with appropriate lymphocytic markers. Langerhans cell sarcoma expresses CD1a, S100, langerin, vimentin, CD68, and BCL1; its histologic features may overlap with those of other histiocytic malignancies. Indeterminate dendritic cell tumor typically involves the skin, sparing the epidermis. The tumor cells resemble Langerhans cells, expressing CD1a and S100, but are negative for langerin, and Birbeck granules are absent. Interdigitating dendritic cell sarcoma features pleomorphic spindle to epithelioid cells that are arranged in sheets or fascicles. The tumor cells are positive for S100, CD68, CD4, CD45, lysozyme, and beta-catenin and lack well-formed desmosomes and Birbeck granules (Fig. 6A-C) [1, 44].

The histological features of secondary MH are similar to those of primary MH. Immunohistochemical staining is essential to confirm the histiocytic origin and distinguish secondary MH from other malignancies. Markers such as CD68, CD163, and lysozyme are typically positive, while markers for the primary malignancy may be absent [43].

Molecular testing is crucial in diagnosing, classifying, and treating MH. Mutations in the MAPK pathway, including *BRAF*, *MAP2K1*, *KRAS*, and *NRAS*, are frequently observed in MH. These mutations lead to constitutive activation of the pathway, promoting uncontrolled cell proliferation. Activating mutations in the *CSF1R*, encoding the colony-stimulating factor 1 receptor, have been identified in histiocytic neoplasms, suggesting a role in disease development and potential therapeutic targeting [18, 40, 41].

### Treatment

Due to the rarity and heterogeneity of MH, standardized treatment protocols have yet to be established, and therapeutic strategies are often individualized based on disease presentation and molecular characteristics [40, 43]. Chemotherapy and surgery have been the most common first-line treatments for multifocal and unifocal disease, respectively. Targeted therapies are administered based on specific molecular aberrations; for instance, patients harboring *BRAF* V600E mutations may be treated with BRAF inhibitors like vemurafenib [43]. Agents targeting immune checkpoints, like PD-1 inhibitors, are being explored, especially in cases exhibiting relevant molecular markers [45].

### Prognosis

The prognosis of MH, like that of many other histiocytic neoplasms, is variable, and is strongly tied to the disease extent and the type of organ involvement. Patients with multifocal disease have a poorer prognosis than those with less disseminated disease [40].

## Primary and Secondary Hemophagocytic Lymphohistiocytosis

HLH is a severe, life-threatening hyperinflammatory syndrome characterized by excessive immune activation, leading to macrophages engulfing blood cells (hemophagocytosis) within organs like the liver, spleen, and bone marrow [46]. It can be familial, due to genetic mutations affecting immune regulation, or acquired, triggered by infections, malignancies, or autoimmune conditions [47, 48].

### Epidemiology

Approximately 25% of reported HLH cases are primary (familial) HLH, predominantly occurring in infants under one year old. The prevalence and incidence of HLH vary widely across regions. Secondary HLH usually presents in children older than 1 year and in adults. The incidence and prevalence of HLH in adults is unclear. In adult HLH, the most common associated conditions have been reported as malignancy (30.7%), infections (24.3%), autoimmune conditions (20.8%), organ transplant (4%), and congenital immunodeficiency syndromes (2.5%) [49].

### Clinical Presentation

HLH is a rare, life-threatening state of immune hyperactivation requiring rapid diagnosis and treatment. HLH presents with a constellation of symptoms driven by an overactive immune response, including prolonged fever, hepatosplenomegaly, cytopenias, hyperferritinemia, hypertriglyceridemia, and hypofibrinogenemia, which indicate severe inflammation and coagulopathy. Severe systemic inflammation may result in altered mental status, respiratory distress syndrome, acute renal failure, or acute liver failure [46, 48].

### Pathogenesis

Primary HLH is a genetically driven immune disorder where the mutations impair cytotoxic function, leading to uncontrolled immune activation and inflammation. Key genes involved in HLH include *PRF1*, *UNC13D*, *STX11*, and *STXBP2*. These genetic mutations impair and disrupt the interaction between CD8 + cytotoxic T cells, antigen-presenting cells, and natural-killer (NK) cells, resulting in the overproduction of pro-inflammatory cytokines. Interferon-gamma (IFN-γ), interleukin-1-beta (IL-1β), and interleukin-18 (IL-18) appear to be the key soluble mediators of HLH immunopathology. This dysregulation activates macrophages, resulting in subsequent cellular destruction [46].

Secondary HLH is commonly triggered by malignancies, infections (especially Epstein-Barr virus), or autoimmune diseases like systemic lupus erythematosus and juvenile idiopathic arthritis, where it is termed macrophage activation syndrome. The interaction between NK and CD8 + cytotoxic T-cell in HLH leads to a fulminant inflammatory response, constantly recruiting additional cytotoxic cells to remove pathologic antigens, resulting in a massive increase in cytokines. Characteristically, in HLH these pathologic, extraneous cytokines promote the further activation of macrophages, resulting in widespread hemophagocytosis and organ-damaging disease [47].

### Diagnosis

Diagnosing HLH relies on a combination of clinical criteria and laboratory findings due to its overlapping symptoms with other inflammatory conditions. The most widely used diagnostic criteria came from the HLH-2004 Diagnostic and Therapeutic Guidelines. Accordingly, the diagnosis of HLH can be established by molecular diagnostic testing consistent with HLH or by meeting five of the following eight criteria: fever, splenomegaly, cytopenias affecting two or more cell lineages, hypertriglyceridemia and/or hypofibrinogenemia, hemophagocytosis, hyperferritinemia, high sIL-2R levels, and low to absent NK cell activity [48].

**Fig. 7:**
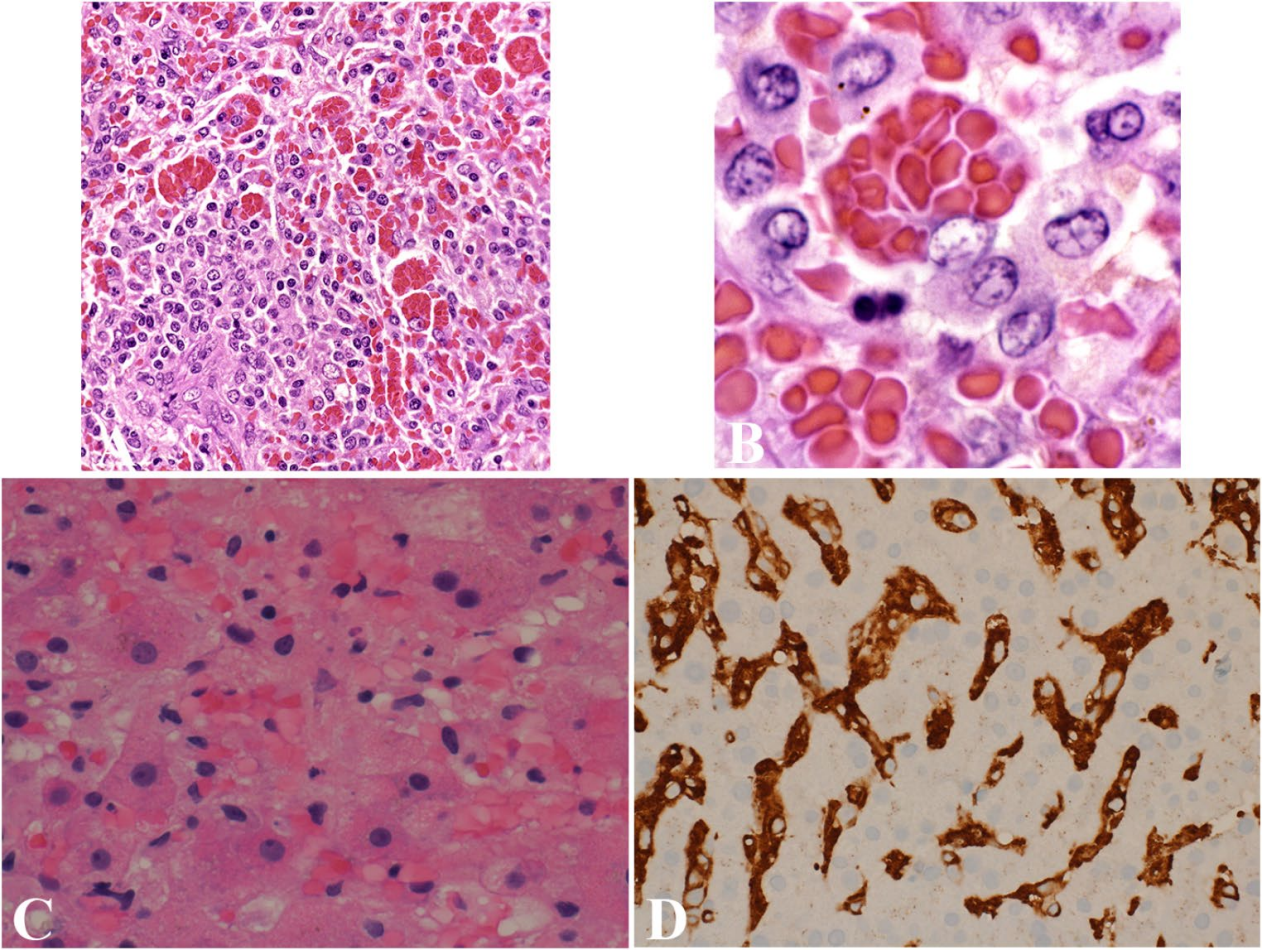
Familial hemophagocytic lymphohistiocytosis. (**A**) A case of familial hemophagocytic lymphohistiocytosis shows numerous histiocytes with engulfed red blood cells. (**B**) High-power view shows histiocytes with numerous engulfed red blood cells. Image from Wang G. Langerhans Cell Histiocytosis, in DP: Molecular Oncology, 3e, by Vasef M, et al. Copyright Elsevier 2024. Reused with permission. (**C**) A liver biopsy shows sinusoidal histiocytic infiltrate with hemophagocytosis. (**D**) CD163 highlights marked sinusoidal histiocytic infiltrates in the liver

Histopathologically, HLH reveals hallmark findings of hemophagocytosis, with activated macrophages engulfing blood cells (Fig. 7A and B) in tissues such as bone marrow, liver (Fig. 7C and D), spleen, and lymph nodes. However, hemophagocytosis is not specific to HLH and may be absent in the early stages, making it a supportive but nondefinitive diagnostic feature. Histopathological evaluation must be correlated with clinical and laboratory findings for accurate diagnosis.

Molecular assays in HLH are used to identify genetic mutations associated with primary HLH. Sequencing of genes commonly associated with primary HLH, such as *PRF1*, *UNC13D*, *STX11*, and *STXBP2*, helps confirm a hereditary basis for the disease [50].

### Treatment

HLH treatment aims to suppress the overactive immune response and manage underlying triggers. Standard treatment typically follows the HLH-2004 Guidelines, which include use of immunosuppressive agents to control inflammation. For primary HLH, hematopoietic stem cell transplantation (HSCT) to replace the defective immune cells is the only curative option. Emapalumab, a monoclonal antibody targeting interferon-gamma (IFN-γ), a key cytokine in HLH pathogenesis, has received FDA approval for pediatric and adult patients with primary HLH who have refractory or recurrent disease or who cannot tolerate conventional therapy [51]. Secondary HLH, triggered by infections, malignancies, or autoimmune conditions, requires both HLH-directed therapy and treatment of the underlying cause [50].

### Prognosis

Without treatment, primary HLH is typically fatal. Early diagnosis and initiation of therapy are crucial for improving outcomes. Since secondary HLH is triggered by infections, malignancies, or autoimmune diseases, the prognosis depends on the rapid identification and management of the underlying cause. Infection-associated HLH, particularly when promptly treated, can have a favorable outcome. However, malignancy associated HLH often presents a more challenging prognosis due to the aggressive nature of the underlying disease [50]. Regardless of etiology, HLH remains a life-threatening condition requiring early recognition and aggressive management to enhance patient outcomes.

## ALK-positive Histiocytosis

Anaplastic lymphoma kinase (ALK)-positive histiocytosis is a rare histiocytic neoplasm characterized by *ALK* rearrangements within histiocytes. This entity was not included in the revised classification of histiocytoses and neoplasms of the macrophage-dendritic cell lineages by the Histiocyte Society in 2016. Now, ALK-positive histiocytosis is a new entity in the 5th edition of the World Health Organization classification of haematolymphoid tumours: myeloid and histiocytic/dendritic neoplasms [52].

### Epidemiology

First described in 2008 [53], this condition has been identified across a broad age range, from infants to adults, and can manifest as either localized or multisystem disease.

### Clinical Presentation

Common clinical presentations include involvement of the liver, hematopoietic system, central nervous system, and skin.

**Pathogenesis**: ALK-positive histiocytosis is driven by genetic alterations involving the *ALK*. Most cases are associated with *ALK* fusions, such as *KIF5B::ALK* or other partner genes. These fusion genes lead to constitutive activation of the ALK protein, even without its ligand. These alterations lead to constitutive activation of oncogenic pathways, including RAS/RAF/MEK/ERK, JAK/STAT, PLC-γ, and PI3K/AKT, resulting in the clonal proliferation of abnormal histiocytes.

**Diagnosis**: Histologically, the neoplastic histiocytes typically exhibit large, pleomorphic, irregularly folded, deeply cleft, or lobulated nuclei, prominent nucleoli, and abundant eosinophilic cytoplasm. These cells may display a spindled or epithelioid morphology and are often intermixed with Touton giant cells and foamy histiocytes. The neoplastic histiocytes exhibit strong expression of ALK protein by immunohistochemistry, and often harbor *ALK* fusions such as *KIF5B::ALK* [54]. Histopathological features overlap with those of other histiocytoses, especially with ECD, RDD, and JXG. Therefore, it is prudent to perform ALK IHC or molecular testing for *ALK* rearrangement in histiocytoses.

### Treatment

Recent studies have demonstrated that patients with ALK-positive histiocytosis respond favorably to ALK inhibitor therapies, leading to significant clinical improvements [55].

### Prognosis

The prognosis for ALK-positive histiocytosis varies depending on the extent of disease involvement, the affected organs, and the treatment response.

## Conclusion

LCH and other histiocytic lesions represent a diverse spectrum of disorders with varying clinical presentations, histopathological features, and molecular characteristics. These conditions pose significant diagnostic challenges due to overlapping histologic and immunophenotypic features. Recent advancements in molecular diagnostics, especially identifying recurrent genetic mutations such as those in the MAPK pathway, have markedly enhanced our understanding of their pathogenesis. These findings have facilitated more precise classification and have provided avenues for targeted therapeutic approaches, particularly in aggressive or treatment-resistant cases.

The role of eosinophils, while prominent in specific histiocytic lesions, reflects underlying inflammatory processes and underscores the complexity of the immune environment within these disorders. However, not all histiocytic conditions are characterized by eosinophilic infiltration, highlighting the need for careful histopathological and clinical correlation in diagnosis. A multidisciplinary approach integrating clinical, radiological, histopathological, and molecular data remains critical for accurate diagnosis and effective management. Collaboration across specialties, including pathology, radiology, and oncology, will ensure comprehensive evaluation and optimized treatment strategies. Ongoing research into molecular pathways holds the promise of novel therapeutic developments needed to improve outcomes in patients with these challenging conditions.

## Data Availability

No datasets were generated or analysed during the current study.
